# Two-Photon Polymerization Metrology: Characterization Methods of Mechanisms and Microstructures

**DOI:** 10.3390/mi8040101

**Published:** 2017-03-27

**Authors:** Christopher N. LaFratta, Tommaso Baldacchini

**Affiliations:** 1 Chemistry Department, Bard College, Annandale-on-Hudson, NY 12504, USA; clafratt@bard.edu; 2 Technology and Applications Center, Newport Corporation, Irvine, CA 92606, USA

**Keywords:** two-photon polymerization, femtosecond laser direct-writing, microfluidics, Raman spectroscopy, nonlinear optical microscopy

## Abstract

The ability to create complex three-dimensional microstructures has reached an unprecedented level of sophistication in the last 15 years. For the most part, this is the result of a steady development of the additive manufacturing technique named two-photon polymerization (TPP). In a short amount of time, TPP has gone from being a microfabrication novelty employed largely by laser specialists to a useful tool in the hands of scientists and engineers working in a wide range of research fields including microfluidics. When used in combination with traditional microfabrication processes, TPP can be employed to add unique three-dimensional components to planar platforms, thus enabling the realization of lab-on-a-chip solutions otherwise impossible to create. To take full advantage of TPP, an in-depth understanding is required of the materials photochemistry and the fabricated microstructures’ mechanical and chemical properties. Thus, we review methods developed so far to investigate the underling mechanism involved during TPP and analytical methods employed to characterize TPP microstructures. Furthermore, we will discuss potential opportunities for using optofluidics and lab-on-a-chip systems for TPP metrology.

## 1. Introduction

Following the success of the microelectronics industry to miniaturize circuits, the scientific community has pursued the goal of reducing their benchtop technologies down to the microscale, with the goal being to shrink the entire laboratory analysis and synthesis capability down to the size of a device that could fit on a microscope slide. This “lab-on-a-chip” idea has become in many ways a reality in the last decade or so [1]. In particular, advances have been made in the fields of microbiology, genetics, and photonics [2,3,4]. Perhaps this is because living cells are naturally at the microscale, so applications involving biology have been the most successful.

When working with chemicals and cells in nanoliter quantities, the norms of macroscopic fluid flow give way to phenomena such as laminar flow, flow focusing, and mixing only by diffusion. The study of fluids at this scale is called microfluidics and has been in existence for quite a long time, but recently the name has also come to be synonymous with lab-on-a-chip (LOC) technology because nearly all LOC devices incorporate small (micron-sized) channels to move around cells and solutions. A microfluidic device may be as simple as a Y-junction channel to mix solutions gradually by diffusion or a massively integrated device with thousands of switches to control the fluid flow of dozens of input and output solutions [5].

What has helped to propel the LOC field has been the development of the technology to create such platforms, namely photolithography. Photolithography uses light and masks to transfer patterns into photosensitive films. Upon exposure through a mask these films become either soluble or insoluble, and after the development stage, patterns are opened on the substrate for further processing, such as metal deposition, doping, or molding [6]. For decades alternative strategies have been investigated included direct laser writing and projection lithography, among many others [7,8,9]. Advances in ultrafast lasers have given way to a form of direct laser writing lithography known at two-photon polymerization (TPP) that has several unique advantages over other forms of lithography [10,11].

The details of the TPP mechanism will be described in this review, but briefly it entails tightly focusing an ultrafast laser pulse into a photosensitive resin and initiating a photochemical reaction at the focal point. Because the laser produces pulses lasing only about 100 fs and because they are tightly focused, the intensity at the focal point is commonly in the TW/cm^2^ regime. These very high intensities enable nonlinear absorption processes to occur within the resin; and since they are nonlinear, the probability of these absorption events falls off quickly away from the focal point. This means that the reaction is highly localized to a single point in space, and can be smaller than the diffraction limit. Structures with arbitrary three-dimensional (3D) geometry can be fabricated by moving the sample with respect to the focal point. Because the resin is typically a negative-tone one, structures are formed in an additive fashion akin to 3D printing. The versatility of design and the extremely high writing accuracy make TPP a powerful tool for the creation of LOC device.

We believe that to use TPP most effectively for applications in LOC devices, it is important to have a clear understanding of the reaction mechanism during fabrication as well as the physical and chemical properties of the finished parts. In this review we will summarize these efforts following two themes: (i) the methods used to characterize the reaction mechanism and (ii) the methods used to characterize a microstructure’s properties. Clearly, the complete mechanism of TPP from photons being absorbed to polymer termination is very complex and with several processes taking place over different time scales, but headway has been made in elucidating the mechanism and we will summarize those recent results. TPP metrology is also a nascent field, with a handful of groups around the world coming up with their own methods to measure these properties. Furthermore, we will speculate of how LOC devices may be employed for TPP metrology.

## 2. Reaction Mechanism

**Figure 1:**
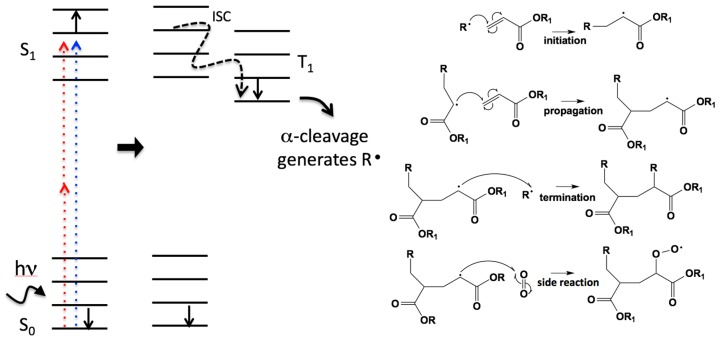
On the left is shown a Jablonski diagram where an electron in the photoinitiator can become excited from the ground state, *S*_0_, to an excited state, *S*_1_, either by absorbing ultraviolet (UV) light or two photons of near-infrared (NIR) light. The excited electron relaxes and flips its spin by internal conversion (IC), and flips its spin by intersystem crossing (ISC), and goes to an excited triplet state, *T*_1_. From *T*_1_ the molecule can undergo α-cleavage to generate radicals, which then begins the polymerization reaction shown on the right. Notably, the last side reaction listed is possible if the resin contains dissolved O_2_. This reaction with O_2_ yields a less labile peroxy radical and effectively quenches the polymerization reaction [19].

The reaction mechanism for ultraviolet (UV) light initiated photopolymerization is well understood and has been studied for decades [12]. The products of photopolymerization have found use in a wide range of applications from adhesives and coatings to microelectronics. The resins used for UV photopolymerization consist of one or more monomers or oligomers and a radical photoinitiator (PI). Other initiator systems exist, such as cationic systems, but here we will focus on radical photoinitiators. The PI can be excited by UV light, but is transparent to longer wavelengths. Upon absorption the PI becomes electronically and vibrationally excited. It quickly relaxes by internal conversion on the time scale of picoseconds, and then undergoes intersystem crossing to a triplet state. Triplet states tend to have longer lifetimes, because their relaxation back to the electronic ground state (*S*_0_) is quantum mechanically forbidden. While in the triplet state the photoinitiator can α-cleave to yield radicals [13]. These chemical species are extremely reactive and will attack carbon-carbon double bonds to produce a new carbon-carbon single bond and another radical species. The new radical species then continues the process growing the polymer chain, which continues until two radical species react terminating the process (Figure 1). Molecular oxygen can also act to terminate the propagation by forming less reactive peroxy radical.

In TPP near-infrared (NIR) light, typically 800 nm, is used instead of UV light to initiate polymerization. The laser pulse, which is generally about 100 fs in duration, goes through a high numerical aperture lens that squeezes the photons in space ultimately resulting in many photons passing through a tiny volume in a very short amount of time. The arrangement makes it probable that two-photons will be absorbed by the same photoinitiator molecule at the “same time”. Under normal conditions (unfocused and non-ultrafast pulses) this is very unlikely because the time overlap for the photons to be simultaneous is on the order of a femtosecond. The electronic jump between *S*_0_ and the first electronic excited state, *S*_1_, corresponds to the energy in the UV, but two photons of NIR light collectively can equal that energy and cause the excitation of an electron. While the localized intensity is quite high and results in two-photon absorption, it is still well below the threshold for self-focusing in the resin. This threshold is on the order of 500 kW, which is about a thousand times higher than the power used in high-repetition-rate oscillators for fabrication and about ten times higher than the power used for low-repetition rate amplifiers [14]. While a variety of laser systems have been used for TPP [15,16,17,18], we focus in this review on the most common system which is Ti:sapphire oscillator with a repetition rate around 80 MHz with energies in the nJ/pulse range. The photophysical steps following two-photon excitation which result in the production of radicals are *assumed* to be the same as that following UV linear excitation. Although this assumption is considered by many to be plausible, to the best of our knowledge no one has studied the radical formation mechanism explicitly for two-photon excitation.

Mueller and coworkers have shown experimentally that after the generation of primary radicals the reaction kinetics of TPP are significantly different from the ones observed in films polymerized by UV irradiation [20]. The difference stems from the large surface area to volume ratio of a typical voxel compared to a film. Diffusion of molecular oxygen, which inhibits radical polymerization, is crucial to the termination reaction in TPP and was found to be the dominant factor in determining the size of the voxel. Experiments were conducted in which a weak continuous wave probe laser was focused through the voxel during TPP and the scattering of this probe beam was measured as a function of time. The change in scattering intensity was assumed to be due to the change in the index of refraction, which increased due to monomer crosslinking. What the researchers found was that following a very short exposure time of 10 µs the polymerization reaction continued in the “dark” and then stopped after a characteristic time, usually about 0.2 ms. They believe this is the time in which the dissolved oxygen consumes all the radicals generated by the exposure. For longer exposures, like 10 ms, the rate of polymerization slows exponentially despite the fact that the laser continues to generate new radicals; however, the time constant for this slowing was found to be strongly dependent on the amount of dissolved oxygen. Again, the amount of oxygen strongly influences the voxel size even for longer exposures. This indicates that quenching by oxygen is more important than radical-radical termination mechanisms which dominate in bulk UV polymerization.

## 3. Nonlinearity Order

An important parameter to measure in TPP is the order of nonlinear absorption, *n*; that is, is it two-photon absorption where *n* = 2, or something else? The value of *n* can be determined by several methods. One is known as *Z*-scan and is used for photoinitiators in solution [21]. To perform a *Z*-scan a thin cuvette containing the PI in a solvent is scanned through the focal point of a simple lens, as opposed to an objective lens, and the transmittance is measured as a function of position. Since the nonlinear absorption is proportional to the intensity raised to the power of *n*, the resulting curve has a different form for different values of *n*. One issue with the *Z*-scan method is that it cannot be performed in the resin. Exposure in the resin results in polymerization, which causes a refractive index change that will confound the change in transmission due solely to absorption.

**Figure 2:**
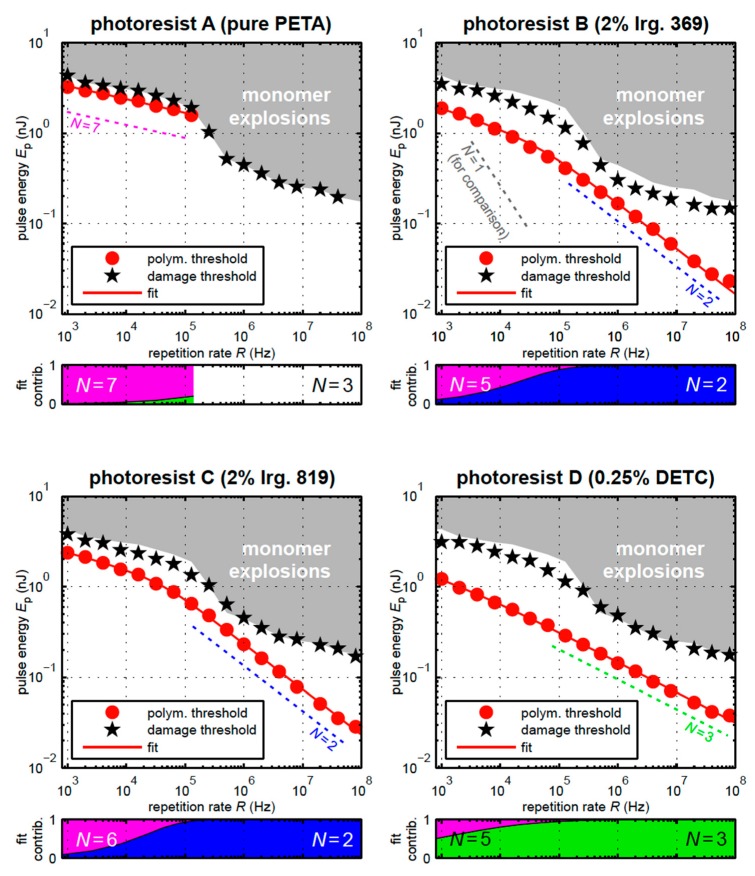
Graphs showing the minimum pulse energy necessary to cause polymerizations (red circles) and uncontrolled polymerizations (black stars) as a function of repetition rate for four different photoresists (**A**–**D**). Under each graph is another graph indicating the magnitude of the weighting factors, such as *a*_2_ or *a*_5_, to the curve fitting in the dashed line above as a function of frequency. Reproduced from [22].

Another method to measure the order of nonlinear absorption, but in the resin, was recently developed by Wegener and coworkers [22]. They used a Ti:Sapphire oscillator delivering 150 fs pulses centered around 800 nm in conjunction with a pulse picker and an acousto-optic modulator to control the repetition rate and pulse energy, respectively. With this setup they were able to vary the repetition rate over a wide range from 1 kHz to 80 MHz. At a particular repetition rate, they adjusted the pulse energy and measured the polymerization threshold above which the polymer would remain after washing, as well as the damage threshold, where uncontrolled polymerization took place. They graphed these thresholds as a function of repetition rate and fit the data to multi-exponential curves, for example: *D*_acc_ = *a*_2_*E*_p_^2^ + *a*_5_*E*_p_^5^. Where *D*_acc_ is the dosage energy accumulated per pulse, *a*_2_ and *a*_5_ are fitting or weight constants and *E*_p_ is the energy of the pulse. The order to which the *E*_p_ is raised corresponds to the nonlinear absorption order, *n*. The authors used the *S*_0_–*S*_1_ energy gap of the photoinitiators as well as the ionization energy of the photoinitiators to inform their data fitting. They found that their monomer, pentaerythritol triacrylate (PETA), could be polymerized without any photoinitiator with a corresponding seventh-order nonlinear absorption, which is approximately equal to the ionization energy of the monomer. Similarly, a resin containing Irgacure 819 as the PI fit the data for a second-order nonlinear absorption, which is nearly identical to the *S*_0_–*S*_1_ gap for that initiator. It was found that as the repetition rate was lowered the order of the nonlinear absorption increased; in most cases from two- or three-photon absorption of the initiator to five-, six-, or seven-photon absorption corresponding to the ionization of the initiator and/or monomer. The magnitude of the weighting factors indicates the transition from low to high order process as the repetition rate decreases, which is shown below each graph in Figure 2.

A different method to measure the order of nonlinear absorption was described by Fourkas and coworkers in a technique they named the two-beam initiation threshold (2-BIT) technique [23]. This method involves splitting an ultrafast laser into two pulse trains, beam #1 and beam #2, and then recombining the pulses with a delay so as to double the repetition rate of the laser. They used a Ti:sapphire oscillator at 76 MHz with pulses approximately 150 fs long centered at 800 nm. The power in each leg after the beam splitter could be independently controlled and the power threshold (*P*_th_) to fabricate a line at 20 µm/s was carefully measured for each leg. To perform the 2-BIT measurement the power of beam #1 (*P*_1_) was made to be a fraction of the polymerization power threshold and then the power in beam #2 (*P*_2_) was adjusted until the polymerization threshold power was reached in the sample. The theory is that a line is form when

(1) $$P_{1}^{n}+P_{2}^{n}=P_{\mathrm{thr}}^{n}.$$

By dividing both sides by $P_{\mathrm{thr}}^{n}$, Equation (1) can be rewritten as

(2) $$P_{1}^{*n}+P_{2}^{*n}=1,$$

where *P*_1_* and *P*_2_* are the laser powers of beam #1 and #2 relative to the polymerization power threshold. Solving Equation (2) for *P*_2_* yields a simple relation between the relative powers:

(3) $$P_{2}^{*}=\sqrt[{n}]{\left(1-P_{1}^{*}\right)}.$$

**Figure 3:**
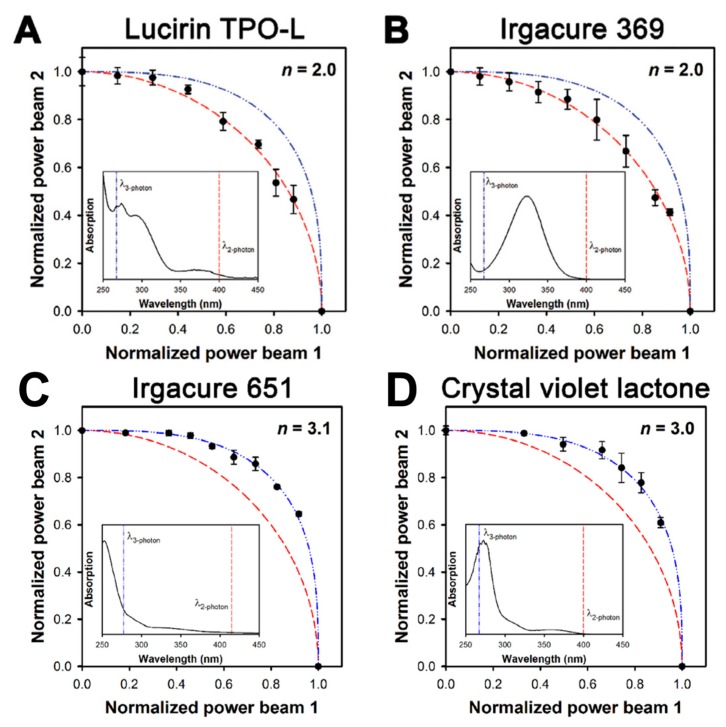
The graphs above show the results of a two-beam initiation threshold (2-BIT) measurement performed on an acrylic monomer resin containing one of the four photoinitiators; (**A**) Lucirin TPO-L; (**B**) Iragcure 369; (**C**) Iragcure 651; and (**D**) crystal violet lactone. The dashed red- and blue-lines correspond to values of *n* = 2 and *n* = 3, respectively. The insets show the UV-vis absorption spectra and the wavelength equivalent of two- or three- photon absorption by the 800 nm excitation laser. Reproduced from [23].

Thus, by plotting the *P*_2_* necessary to reach the polymerization threshold for different values of *P*_1_* and fitting the data with Equation (3), the values of *n* can be measured as shown in Figure 3. The researchers performed the 2-BIT measurement for four different photoinitiators in an acrylic resin; TPO-L, Irgacure 861, Irgacure 651, and crystal violet lactone. The latter two photoinitiators were determined to start photopolymerization by three-photon absorption while the first two photoinitiators were determined to start photopolymerization by two-photon absorption.

There are two assumptions that need to be met for the 2-BIT method to be effective. The first is that the time between pulses is long enough to ensure radicals generation. This condition is met easily since the time to generate radicals is on the order of 100 ps, and the time between two consecutive pulses is a few nanoseconds. The other is that time between pulses is short enough so that diffusive effects are negligible.

Comparing the three methods to measure the nonlinear order, the *Z*-scan, the variable repetition rate, and 2-BIT, they each have their strengths and weaknesses. *Z*-scan is relatively straightforward to perform but cannot be run in a resin, which severely limits its utility. The variable repetition rate method requires a somewhat more complex experimental setup than a typical one used for TPP, but it can provide data that spans different photochemical regimes from photoionization to TPP. Requiring only beamsplitter and power control optics, the 2-BIT method can be readily assembled in any laboratory capable of TPP, but does not enable the multi exponential fitting like the variable repetition rate method. Although the method one choses will likely be determined by the equipment available in one’s laboratory, it is likely that the 2-BIT method will find wider use because of the simplicity of its implementation.

## 4. Thermal Effects

The effect of temperature on the photopolymerization mechanism has long been of interest starting with a paper by Kawata and coworkers in 2008 [24]. In that paper the entire sample was thermally regulated from −50 to 50 °C and TPP linewidths were measured. It was found that at low temperature the linewidths got smaller because diffusion was limited; however, it was also found that above room temperature the linewidths also got smaller. The authors explained that this could be due to the well-known effect of temperature accelerated termination of the polymerization reaction.

In more recent works, the actual temperature change during TPP was measure by Mueller and coworkers [25]. They used up-converting nanoparticle luminescence, which is temperature-dependent, to measure the temperature at the polymerization focal point in situ. Their results showed a very modest temperature increase of about 5 °C under normal laser writing conditions with a Ti:sapphire oscillator. At higher powers, when micro explosions occurred, they measured much larger temperature changes, in the hundreds of degrees. The conclusion was that temperature changes under typical TPP writing conditions do not have much of an effect on the polymerization process because the temperature change is so subtle.

One of the authors of this review has performed a different experiment that indicated a heating effect that resulted in larger linewidth when laser pulse bursts were closely spaced [26]. The experiment took an ultrafast pulse train and chopped into 1 µs bursts with variable time between bursts. When the bursts were close together linewidths were larger than when the bursts were spaced further out in time. This indicates that heat could not dissipate between the bursts and its buildup resulted in larger lines. The threshold time between bursts where this effect became noticeable corresponded well with the material cooling time of the resin, 16 µs.

The effects of thermal accumulation on TPP have been investigated also by using a diode-pumped picosecond Nd:YVO laser with cavity dumping. Specifically, the fundamentals at 1064 nm and the 532 nm frequency-doubled wavelengths were used for fabrication using laser pulses ranging from 8 to 25 ps and repetition rates from 0.2 to 1 MHz [16]. The authors of this study observed that thermal accumulation becomes significant and influence TPP only when the cooling time of the irradiated spot becomes comparable to the time separation between consequent pulses.

## 5. Characterizing Sizes, Shapes, and Surface Roughness

The ultimate complexity and reproduction fidelity of three-dimensional microstructures fabricated by TPP depends greatly on factors influencing the shape and size of the smallest polymerized volume element or voxel. Experimental parameters that are employed to adjust the voxels’ dimensions are the laser power and exposure time, the numerical aperture of the focusing lens, and, to a lesser extent, the polarization of the excitation light [27,28]. It is the accurate overlapping of voxels that produces the desired geometry and surface roughness in the final structure. Thus, the characterization of voxels is a fundamental step in TPP; one that can have great consequences on the success of the whole fabrication process.

Samples used to determine TPP voxels’ sizes are prepared by means of either the single voxel method or the single line method. In the single voxel method, a stationary laser beam creates a single voxel. By adjusting the laser average power and exposure time, voxels with different lengths and diameters are created. In the single line method, a polymerized line is fabricated by moving the sample at constant velocity around a fixed laser beam. Lines with different dimensions are created by adjusting the laser average power and the sample scan velocity. Although the two methods are equivalent in the information they provide because of the reciprocity between laser exposure time and scan speed, the line method is preferable. Since TPP microstructures are formed by overlapping continuous trajectories (lines) in all three dimensions, the line method correlates more directly writing conditions to TPP feature sizes than the single voxel method.

**Figure 4:**
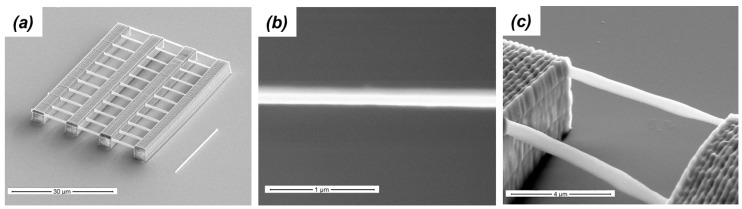
Scanning electron microscopy (SEM) images two-photon polymerization (TPP) suspended lines made with a single laser pass. While the complete structure is shown in (**a**), the top and side views of one of the lines are shown in (**b**,**c**), respectively. The scale bars are 30, 1, and 4 µm for (**a**–**c**), respectively.

A typical experiment employed in TPP to determine how experimental writing conditions influence the overall dimensions of polymerized lines is shown in Figure 4a. A group of parallel lines each fabricated using only one laser pass is written across two large supports that are anchored to the substrate. Since the fabrication of these lines take place far from the substrate/resin interface, the extent of the polymerization confinement in space is completely revealed. The lateral and axial dimensions of a fabricated line are measured by means of scanning electron microscopy (SEM) where images are recorded with the sample normal and at 45°, respectively (Figure 4b,c). When the suspended lines are made using different writing speeds and laser average powers, samples such as the one in Figure 4a are used to produce calibration curves where polymerized line dimensions are plotted versus experimental writing conditions [29]. It is the information retrieved from these calibration curves that permits the exquisite three-dimensional writing that has rendered TPP unique as an additive micromanufacturing technique.

The line method described so far is particularly effective when using low-shrinkage resins such as hybrid organic-inorganic photosensitive materials, SU-8, and acrylic-thiol mixtures. It becomes less adequate when using resins that undergo substantial shrinkage upon polymerization such as acrylic based resins. In this case, the numerical results of the line method are a combination of the material shrinkage and the material response to the different writing parameters. To minimize the effect of the material’s shrinkage in the measurement of the polymerized line’s final dimensions, a modified version of the line method is applied. The series of lines are written directly at the substrate-resin interface and then imaged by SEM. Because they are anchored firmly to the substrate, such lines exhibit less shrinkage than the analogous one written in the resin volume.

The main disadvantage of this modified version of the line method is the setting of the optimal *z*-position within the substrate where to perform the writing of the test sample. To avoid mistakes in locating the proper *z*-position, every pattern is repeated for different *z*-positions. Imaging by SEM is then used to identified the pattern that was written at the optimal *z*-position which corresponds to the pattern that does not present lines fallen over. Another disadvantage of the modified line method is its inability at measuring the axial dimension of the written line.

**Figure 5:**
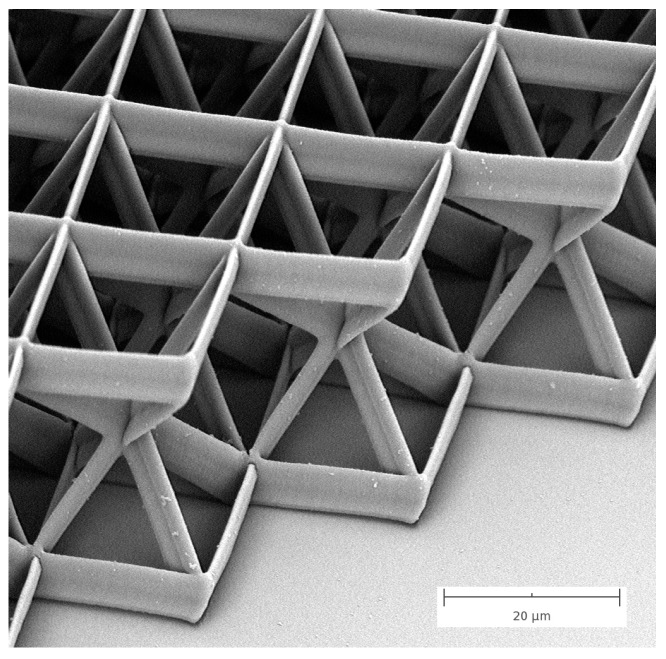
Example of imaging of a TPP microstructure complex geometries by SEM. Scale bar is 20 µm.

High resolution SEM is without any doubt the best way to measure feature sizes in TPP. It is a fast, non-destructive imaging technique which requires little or no sample preparations and it delivers images with more than adequate spatial resolution for precise and repeatable measurements. Furthermore, imaging by SEM is also tremendously effective at capturing the topology of complex three-dimensional microstructures fabricated by TPP. By rotating and tilting the sample, imaging by SEM enables direct views of three-dimensional architectures that would be otherwise impossible to perform with other imaging techniques. An example of this ability is displayed in Figure 5, where the SEM image of a microstructure fabricated by TPP is shown. The composition of the structure unit cell with the four diagonal beams is clearly exposed in this image.

Although imaging microstructures by SEM is an effective way to characterize TPP, it is not suitable for measuring surface roughness. This property is critical in micro-optics applications such as whispering gallery micro-cavities and micro-optical elements where the surface roughness of microstructures needs to be several times smaller than the wavelength of the testing light. Accordingly, scanning probe techniques like atomic force microscopy (AFM) are in a better position than SEM to quantify surface roughness of TPP microstructures. These surface imaging techniques are sensitive to sub-nanometer height differences, enabling the measurement of surface roughness with great precision. By choosing the proper writing conditions, several research groups have fabricated polymeric optical components with surface roughness down to few nanometers as measured by AFM [30]. Thus, the optical quality of TPP microstructures can be made smooth enough to be acceptable for application as optical devices in the visible and near-infrared region of the spectrum.

## 6. Dealing with Shrinkage and Surface Tension

Among the several type of negative-tone resins used in TPP, the ones based on multifunctional acrylates have received considerable attention. This is not surprising if one takes into consideration the favorable attributes acrylic monomers and oligomers possess [31]. Since they are widely used in several industries, acrylic molecules are inexpensive and readily available. They have a relatively stable shelf life, and can be found in a wide assortment of functionalities and sizes. In their un-polymerized state, acrylic resins are soluble in common solvents such as ethanol and they are easily processed by spin-coating or drop-casting. Furthermore, acrylic moieties retain high polymerization rates due to their reactivity toward active species such as radicals.

Despite the advantages acrylic monomers and oligomers impart to resins used in photopolymerization, they also present some limitations [32]. Specifically, acrylic resins are known in producing inhomogeneous polymer networks and they are sensitive to the presence of dissolved molecular oxygen. Perhaps, the most aggravating disadvantage of using acrylic resins in TPP is the degree of shrinkage they undergo during polymerization.

Shrinkage occurs because of volume contraction during polymerization [33]. In this process the intermolecular separation between monomers is reduced to shorter distances as a result of the formation of covalent bonds. The extent of volume shrinkage developed during polymerization depends on many factors such as the molecular structure of the monomers and the degree of material conversion or fraction of monomers converted to polymer. Shrinkage can reach values as high as 20% in the polymerization of acrylates and methacrylates. Thus, microstructures fabricated by TPP suffer greatly from shrinkage where stresses generated during polymerization can develop internal and/or interfacial defects as well as considerable deformations.

To eliminate or at least mitigate the complications derived by shrinkage during TPP, several approaches have been implemented. In one, a pre-compensation scheme is used during the writing of microstructures so to cancel out deformations developed during polymerization [34]. In another one, microstructures are fabricated on top of multi- and single-anchor supports to isolate them from the substrate [35]. In this approach, shrinkage still occurs but in an isotropic way thus producing three-dimensional objects with the correct proportions between height, length, and width.

In addition to these methods, novel materials that intrinsically produce polymers with low shrinkage have been developed for TPP. One of such resins is based on a zirconia sol-gel process that yields an organic-inorganic material with favorable characteristics [36,37]. Specifically, microstructures fabricated out of this resin exhibit negligible shrinkage (<1%) when using high average laser powers. In this case, microfabrication requires first a prebaking step during which a condensation process creates a solid film made out of a rigid inorganic skeleton. Then, laser structuring is used to polymerize the organic part of the film forming an insoluble crosslinked network. TPP is used essentially to connect pendant methacrylic moieties attached already to a solid framework. Hence, the polymerization of this resin that occurs via two consecutive steps forms an almost a non-shrinkable material leading to distortion free microstructures.

A different chemical approach was recently demonstrated where the authors took advantage of thiol-ene chemistry [32]. It is well-known in the UV curing of acrylic and methacrylic films that the addition of multifunctional thiol molecules enhances several aspects of the polymerization process. Furthermore, it produces films with improved mechanical and chemical properties. For example, the shrinkage associated with the polymerization of (meth)acrylates is 22–23 cm^3^/mol per reacted double bond, while it is between 12 and 15 cm^3^/mol in the case of thiol-(meth)acrylate polymerization. The ability of thiol molecules to reduce volume shrinkage in the polymerization of acrylic resins stems from the observation that the co-polymerization of these monomers develops a cross-linked network with a high gel-point conversion. Any shrinkage arising prior to the gel-point formation can easily be contained by viscous flow adjustments.

**Figure 6:**
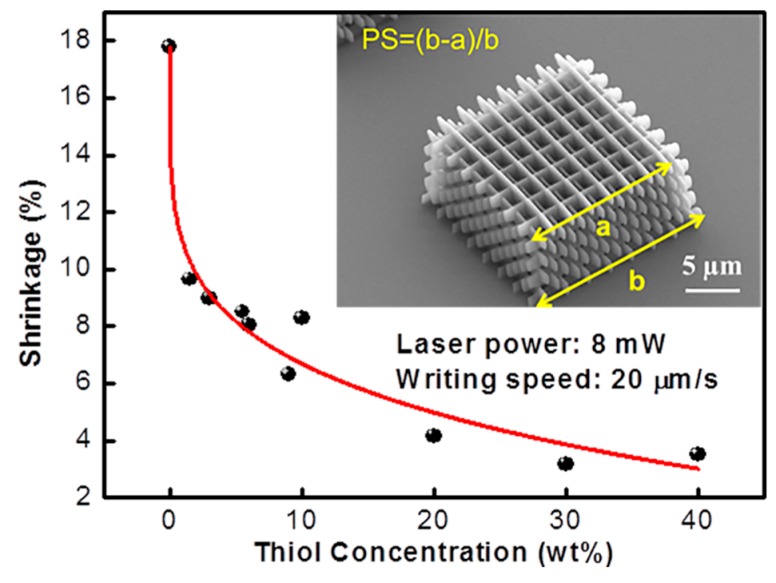
Shrinkage of a TPP microstructure using acrylic resins with different concentrations of multifunctional thiol molecule. The red line is used as a guide to the eye. A SEM image of the microstructure with the definition of percentage of shrinkage (PS) used in this study is shown in the inset. Reproduced from [32].

To confirm this improvement imparted by the use of thiol molecules in (meth)acrylate resins in TPP also, the authors of the mentioned work fabricated a series of microstructures with resins containing different amount of a multifunctional thiol molecule. The test sample consisted of a 16-layer woodpile structure. All samples were made under the same experimental conditions. The percentage of shrinkage (PS) was extrapolated by SEM images of the microstructures measuring the length difference between the top and bottom layers of the woodpile structures. Figure 6 shows that the shrinkage of the unadulterated acrylic resin is around 18%. As the concentration of the multifunctional thiol molecule in the resins reaches 10% in weight, the shrinkage of the microstructure drops of more than half from its original value to around 6%. A further increase in the thiol concentration produces only a minor improvement, with shrinkage approaching 4% at a multifunction thiol molecule concentration of 40%.

Although shrinkage is often deleterious toward TPP microstructures, it can also be used as an advantage in specific applications. For example, three-dimensional photonics crystals have been made freestanding by TPP. Because of isotropic shrinkage, the whole microstructure resulted smaller than the analogous fabricated attached to the substrate. Hence, shrinkage was used to make photonic crystals with band gaps shifted toward shorter wavelengths [38].

The maximum precision in microfabrication by TPP is achieved when working at close-to-threshold conditions. The smallest and more symmetric voxels are achieved when using laser average powers barely above the value necessary to start and sustain polymerization. Unfortunately, these are also the weakest voxels from a mechanical point of view since they are created with small polymer degree of conversion. As a consequence, microstructures fabricated with these voxels tend to buckle and collapse under the pressure of surface tension during the drying process that follows the developing step. This is the case especially for microstructures with high-aspect ratio and/or with complex and densely packed geometries.

Mechanical failure during the development step occurs when the liquid cohesive forces are larger than the polymer restoring forces. The cohesive forces arise from the receding level of the solvent/resin liquid mixture which forms a concave meniscus between the features of the solid microstructures. As a consequence, during draining, the entire polymeric object feels pulling forces that can cause bowing of some of its parts. Because of the size of the microstructures created by TPP, these forces can be quite large. In the case of two parallel walls, for example, the cohesive force is directly proportional to the liquid surface tension and inversely proportional to the distance between the walls.

There are two strategies to overcome the defects generated by surface tension of the resin rinse during the developing step. The first one is to make the polymeric microstructure stronger (i.e., increasing the restoring forces) [39]. For example, it is common to polymerize only the shell of a complex microstructure in order to optimize fabrication time. Making the wall thicker by means of a multi-path laser design is a good way to ensure that it survives intact the developing step. This is because the mechanical strength is increased cubically with an increase of thickness. The second strategy instead, tends to remove the effects of surface tension all together. This is accomplished by performing the rinsing step using supercritical CO_2_ drying [40]. Since supercritical fluids have properties similar to both gases and liquids, they do not have a gas–liquid interface, thus eliminating the damaging effects of surface tension. This second method is preferable in the extraction of complex and fragile microstructures that need to be made with single-path laser designs.

## 7. Measuring Mechanical Properties and Adhesion Characteristics

TPP microstructures are used in several applications where their structural integrity or their ability to comply to external stimuli is important. In microfluidics for example, polymeric microstructures can be used as filters for sorting or extruding cells [29]. To ensure the correct operation of these devices, it is critical that the structure of the filter remains intact even at high flow rates, and that, during operation, the filter itself does not get dislodge from the substrate. Thus, several analytical methods have been utilized to extrapolate information on the mechanical properties of microstructure fabricated by TPP.

Interpreting the results of structural experiments can often be ambiguous, since several factors contribute to the mechanical performances of TPP microstructures. Increasing laser average power for example, produces voxels with higher degree of conversion (i.e., stiffer). At the same time, the size of the voxels grows as well. Accordingly, the amount of voxels overlap during microfabrication is different for different laser average powers. In this situation, the microstructure will become stronger as the laser average power used to make it is increased. Both factors (degree of conversion and voxels overlap) drive this improvement, but it is difficult to separate their contributions and quantify their relative importance. Thus, measuring mechanical properties of TPP microstructures requires taking into consideration not only the photophysical process of TPP but also the writing scheme employed in the microfabrication.

In the first report describing the mechanical properties of TPP microstructures, an optical tweezer scheme was used to stretch a polymeric coil with a spiral radius of 150 nm that was attached to a solid polymer anchor on a glass substrate [41]. The experiment was performed with the sample immersed in an organic solvent. By observing the spring recovery from its elongated position, the authors were able to infer the material’s shear modulus. The shear modulus of the TPP microstructure was significantly smaller than the shear modulus of the bulk polymer (0.5 MPa vs. 150 MPa). Although a full understanding of this surprising result is not yet available, two factors are the more plausible causes [42]. The first is the effect of solvent permeation, which could reduce the polymer elasticity [43]. The second is a reduction of the polymer glass transition as its dimensions are shrunk to hundreds of nanometers [44].

A contrast between the mechanical properties of TPP microstructures and of bulk polymer was observed also when using a different analytical method, although this time the gap was much smaller. In this case, a commercial atomic force microscope (AFM) with a calibrated cantilever was used to perform force–distance measurements on polymeric beams made by TPP [43]. The microstructures Young’s modulus (*E*) was retrieved by their spring constants (measured by AFM) and physical dimensions (measured by SEM). The results for E were 0.4 and 2 GPa for the TPP microstructures and the bulk polymer, respectively. This time, the discrepancy in stiffness is believed to be caused by the fact that TPP microstructures are polymerized on a voxel-by-voxel basis while the bulk polymer is formed by UV flooding illumination. This study has shown also that the relatively low elastic modulus of TPP microstructures makes them attractive candidates for contact measurements of soft condensed matter.

A favored method among scientists and engineers for measuring mechanical properties is nanoindentation. This is because it provides both elastic modulus and hardness with high precision and reliability. Furthermore, it is the most accommodating technique for probing mechanical properties of structures at the micro and nano scale. Nanoindentation has been successfully used on TPP microstructures. In one case for example, the Young’s modulus of a commercially available resin was measured by performing stress–strain tests on TPP micro pillars [45]. Where nanoindentation plays a critical role is in the determination of mechanical properties of materials with controlled microstructural architectures. These are materials that can be designed with low densities and high strength, and as such can be used in several technologies from energy storage to sensing. Since nanoindenter probes can be integrated within the chamber of SEMs, phenomena such as failure, deformation, and recoverability can be analyzed and visualized in real time and in situ by performing compressing tests using flat punch tips [46,47].

**Figure 7:**
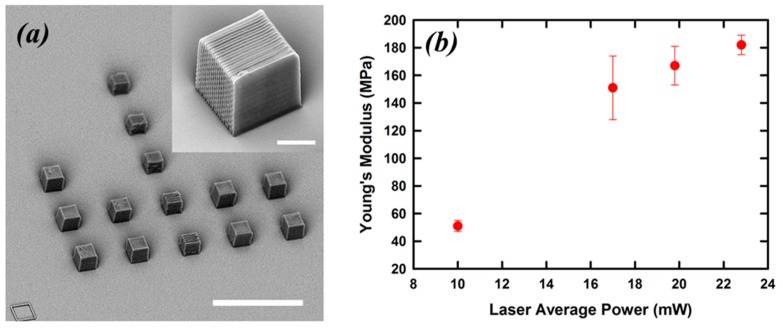
SEM of the TPP microstructures used in the mechanical testing (**a**); scale bare is 40 µm. A close-up image of one of the tested cubes (**b**); scale bar is 5 µm. Dependence of microstructures’ Young’s modulus on laser average power used to make them (**b**). Reproduced from [29].

Besides nanoindentation, another method particularly suited to study the mechanical properties of TPP microstructures is the one that uses MEMS-based force sensing probes. These machines consist of multi-directional tools to measure or manipulate objects at the micron scale. They are capable of performing nN to mN range force–position–time measurements in compression, tension, and adhesion tests. In a recent example, this technique was used to retrieve the Young’s modulus of an acrylic-based resin polymerized by TPP [29]. The test sample was an array of polymeric cubes with sides measuring 10 µm (Figure 7a). The cubes were fabricated layer by layer by overlapping a series of parallel lines with a spacing of 0.5 µm. The distance between each layer was 0.5 µm as well. All cubes were written using a scan speed of 20 µm/s, and the laser average power was varied from 10 to 23 mW. Compression measurements were performed by approaching the cubes from the top with the MEMS-based force probe. Upon contact with the polymeric sample, the probe pushed on it and recorded both the force applied and the position, which in this case is related to the sample deformation. The material’s Young’s modulus is measured by using the sample stiffness and the sample’s dimensions.

The results of these measurements are shown in Figure 7b, where the retrieved Young’s moduli are plotted versus the laser average power. The range of modulus values overlapped well with values expected for acrylic based polymers. A rapid increase in the *E* from 52 to 185 MPa is observed when the laser average power is raised from 10 to 23 mW. A separate test was performed on a different sample that took into consideration the polymerized line width variation when using different laser average powers. Specifically, the new sample was fabricated by changing the hatching between the lines in *XY* plane and the between the layers in the *Z* axis so to maintain the same degree of lines/layers overlap independently of the laser average power used. The result of this test showed that the main contributor to the Young’s modulus of the samples in Figure 4a is the degree of polymer cross-linking achieved using different laser average powers.

**Figure 8:**
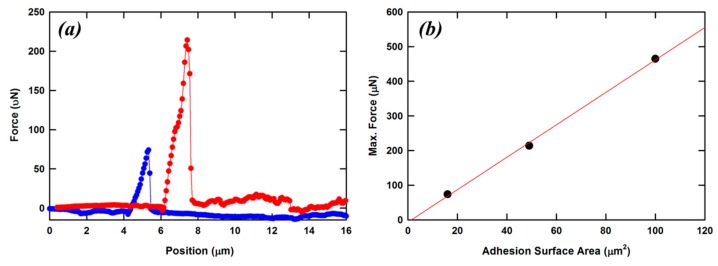
Lateral force measurements of cubes fabricated by TPP onto glass substrate (**a**). Forces applied to a cube with the side 4 μm long are represented with circles; forces applied to a cube with a side 7 μm long are represented with triangles. The solid lines are intended only as a guide to the eye. Maximum force required to dislodge a polymer cube from its substrate is a function of the contact surface area (**b**). The solid line is a linear regression. Reproduced from [29].

By employing a lateral probe in the same testing machine, the authors of the previous work were also able to measure the adhesion properties of TPP microstructures. The sample consisted again of a series of polymerized cubes with sides of 10, 7, and 4 µm. The writing speed and the laser average power were not changed during the fabrication. Since all cubes were made using the same writing scheme and the same experimental conditions, the expectation was that the adhesion properties of these objects must reside in the surface area which is in contact with the glass substrate. The force displacement curves for two cubes are shown in Figure 8a. No significant force is measured before contact between the sample and the probe. As soon as contact is engaged, the applied force increases with minimum displacement of the microstructure. When the applied force exceeds the adhesion between the cube and the glass, the probe will measure again displacement with no force. This is because the cube is no longer attached to the glass substrate. The maximum force needed to dislodge the cube from the glass substrate were measured and plotted as a function of the surface contact area in Figure 8b. A clear linear dependence is observed with a slope of 4.7 µN/µm^2^.

## 8. Investigating Chemical Properties

Several physical and chemical properties of polymers are a direct consequence of the material degree of conversion (DC). In general, DC is the percentage of starting material such as monomers and oligomers that have been covalently linked together to form the product, the polymer. In the specific case of resins made of acrylic monomers, DC is the percentage of ene moieties polymerized into one interconnected chemical structure.

The measurement of polymer DC as excitation and/or environmental conditions are varied, is a critical step in determining potential uses of photopolymers. For example, in biological applications, materials need to be polymerized with the highest DC since any leaching of unreacted molecules in the surrounding environment might be deadly to cells and tissues. Furthermore, it was recently shown that in the fabrication of micro-optics, knowledge and tailoring of the polymer DC can be used in the formation of graded-index (GRIN) elements [48]. Numerous methods have been used to extrapolate DC values of polymers such as differential scanning calorimetry (DSC), Fourier transform infrared (FTIR) spectroscopy, and Raman spectroscopy [49,50]. Although DSC is the industry standard for characterizing DCs, it is not well suited for testing TPP microstructures. This thermo-analytical technique has a stringent minimum weight requirement and causes irreversible damage to the samples. Poor spatial resolution and distinct sample preparations are among the drawbacks of FTIR spectroscopy. Raman spectroscopy instead, is an optimal choice for the static characterization of microstructures fabricated by TPP [51]. It allows for in situ and nondestructive monitoring of specific bond vibrations with excellent spectral resolution and minimum sample preparation.

Raman spectroscopy provides comprehensive information about the vibrational modes of molecules by using light scattering. The Raman-scattered light from the molecules in a probed sample is shifted in the frequency domain relative to the excitation light center frequency, and it contains information about the frequencies and anisotropies of molecular vibrations. Vibrational modes are like fingerprints of molecules, rendering Raman scattering a unique tool for discerning different molecular species with high sensitivity. Consequently, Raman spectroscopy has broad applications, ranging from basic research in chemistry, biochemistry, art, and archeology to highly practical forensic examinations.

As already mentioned, acrylic-based materials are commonly employed in TPP. To illustrate the utility of Raman microspectroscopy in characterizing TPP it is thus useful to briefly describe the polymerization process involved. Acrylic-based resins are composed mainly of two molecular components, a photoinitiator and a mixture of monomers/oligomers. The first one is the molecule capable of undergoing excitation by two-photon absorption and of generating active species (radicals) that start the polymerization process. In the presence of excited photoinitiators, the monomer/oligomer mixture forms a complex linked network, a polymer. Monomers and oligomers are present in the resin in much larger numbers than photoinitiators.

Radicals generated during laser irradiation react with the monomer/oligomer mixture to form high molecular weight materials through a chain growth mechanism. Several reactions with different rates for initiation, propagation, chain transfer, and termination occur at the same time. In chain polymerization, monomers and oligomers react only with the propagating reactive center, not with other monomers and oligomers, and chain addition ceases when the active species are depleted by a number of termination reactions. The molecular weight of chain polymers increases rapidly during polymerization and monomer/oligomer to polymer conversion (degree of conversion) can widely range between 20% and 90% depending on the chemical nature of the monomers and oligomers. In acrylic monomers and oligomers, chain addition takes place in the carbon-carbon double bonds (C=C) present in molecules ester moiety. Thus, during TPP the number of C=C bonds diminishes in favor to the formation of new inter-molecular carbon–carbon single bonds (C–C).

**Figure 9:**
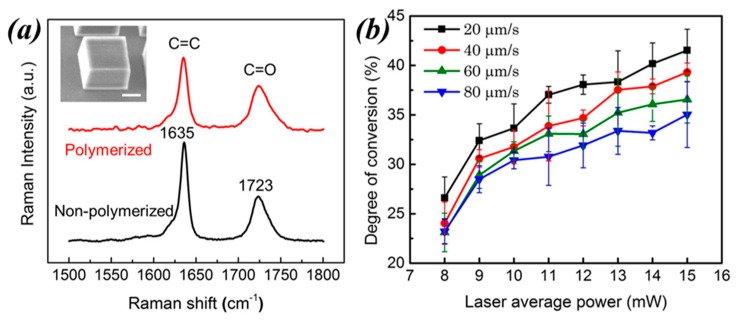
(**a**) Raman spectra of polymerized and unpolymerized acrylic resin. The spectra are shifted vertically for viewing clarity. The inset is a SEM of a tested TPP microstructure (scale bar is 5 µm). (**b**) DCs of TPP microstructures made with different laser average powers and scanning speeds. Reproduced from [51].

The Raman spectra of a typical resin employed in TPP before and after polymerization are shown in Figure 9a. An example of the TPP microstructure used in this study is shown in the inset of Figure 9a. Two distinctive peaks are observed at 1635 and 1723 cm^−1^ which are ascribed to the vibrational modes of C=C and the carbonyl (C=O) groups of the monomer, respectively. As a consequence of the diminished concentration of the C=C bonds following polymerization, the intensity of the peak at 1635 cm^−1^ in the polymerized resin is smaller than in the unpolymerized resin. On the other hand, the intensity of peak at 1723 cm^−1^ remains the same before and after polymerization because the carbonyl group does not participate in the chain reaction.

The area of a peak in a Raman spectrum is proportional to the concentration of the oscillators responsible that particular Raman active mode. Thus, spectra such as the ones in Figure 9a can be used to measure the DC in resins following TPP. DC represents the number of C=C bonds consumed during polymerization. Since the number of carbonyl groups in the resin remains the same before and after polymerization, the peak at 1723 cm^−1^ can be used as internal reference for DC estimation. The percentage of DC is obtained using the following equation:

(4) DC=[1−AC=CAC=OAC=C′AC=O′],

where *A*_C=C_ and *A*_C=O_ are the integrated intensities of the 1635 and 1720 cm^−1^ peaks in the polymerized resin, respectively, while *A’*_C=C_ and *A’*_C=O_ are the integrated intensities of the same peaks in the unpolymerized resin.

To evaluate the usefulness of Raman spectroscopy in TPP, a series of micro-cubes were fabricated first and then characterized using a confocal Raman micro-spectrometer. An SEM image of a microstructure used in the study is shown in the inset of Figure 9a. The micro-cubes were fabricated using several laser average powers and writing speeds. For each microstructure, Raman spectra like the ones in Figure 9a were recorded and then used to measure DCs using Equation (4). The results are shown in Figure 9b. At constant writing speeds, DC values monotonically increases with increasing laser average power. The highest DC value is around 43%; larger DCs were not attainable because the resin started to boil if higher laser average powers were used.

The maximum DC is ascribed to the branched nature of the acrylic monomer. During polymerization, crosslinked oligomers are formed that are difficult to diffuse freely. With increasing DCs, the mobility of the oligomers is so limited that further polymerization process is terminated, therefore limiting the maximum DC to values smaller than 50%. The phenomenon of incomplete conversion of resin into a polymer is common when using highly branched acrylic monomers [52].

To examine whether or not the polymerization initiation mechanism has an effect on the resulting polymer DC, the acrylic-based resin used to make the structures employed in Figure 9a was polymerized using a conventional UV light source as well. Investigation of this sample by Raman spectroscopy revealed a maximum DC value of 45%, which coincides well with the maximum DC value obtained by TPP.

There has been a lot of research on the materials side in order to increase the number of available resins for TPP. A class of resins that is becoming more and more popular is the one based on inorganic–organic hybrid polymers. This is due a series of advantages they possess over more conventional pure organic resins. Specifically, they have superior chemical, mechanical, and thermal properties. Consequently, Raman spectroscopy was used to characterize TPP microstructures made out of these materials [48,53]. The results have shown DC values as high 80%.

## 9. Imaging by TPEF and THG Microscopy

Two-photon excited fluorescence (TPEF) microscopy is a three-dimensional imaging technique capable of peering deep into scattering samples with submicron resolution. By employing near-infrared femtosecond lasers and optimally designed fluorophores, TPEF microscopy has been used to study complex cellular events in vivo at depths exceeding few millimeters, thus becoming an invaluable tool in several biomedical applications [54].

**Figure 10:**
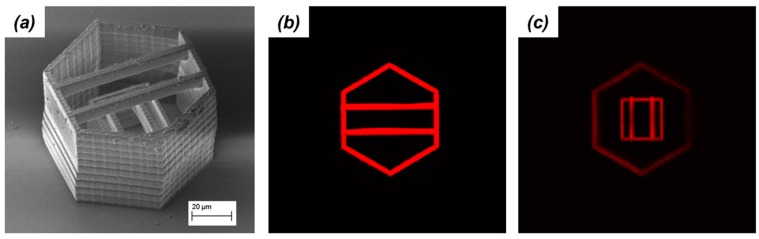
(**a**) SEM image of two TPP microstructures doped with Rhodamine B. The smaller structure was written inside the larger one and so difficult to observe (scale bar 20 µm). Two-photon excited fluorescence (TPEF) microscopy images of the TPP microstructures recorded at different heights are shown in (**b**,**c**). They reveal two *z*-sections of the sample with no cross-talk.

TPEF microscopy can be used to image three-dimensional microstructures fabricated by TPP as well. Although many resins employed in TPP display a certain amount of autofluorescence after polymerization, the strength and homogeneity of this signal are not high enough to produce clear images a low excitation powers. By choosing a fluorophore that does not interfere with the action of the photoinitiator, a fluorescent-doped resin can be used in TPP producing microstructures that can easily be interrogated by TPEF microscopy. An example is shown in Figure 10. An acrylic-based resin containing a small amount of Rhodamine B was used to create a complex microstructure by TPP. Rhodamine B is a fluorophore that upon excitation by two-photon absorption emits a broad fluorescence with maximum intensity at 610 nm. Upon polymerization, the dye molecule is entrapped in the polymer, leading to highly fluorescent microstructures.

The microstructure consists of two towers, one inside the other; a large one with a hexagonal base and a smaller one with a square base. At the top of each microstructure, two freestanding beams connect adjacent sides. Since the outside tower is 40 µm tall while the inside tower is 20 µm tall, there is no overlap among the two sets of beams. As the image in Figure 10a shows, it is difficult to clearly expose all the parts of the microstructure by SEM. When imaging the same microstructures by TPEF microscopy instead, cross-sections can be retrieved with high spatial resolution. The fluorescent image recorded at a height of 30 µm from the substrate (Figure 10b) shows the framework of the hexagonal tower and the horizontal beams. The fluorescent image recorded at a height of 8 µm from the substrate (Figure 10c) shows strong signal from the square tower and the vertical beams. A faint signal from the hexagonal tower is observed also in this figure. No signal crossover is observed in the two fluorescent images.

Hence, TPEF microscopy is a complimentary tool to SEM in the diagnosis of TPP microstructures. For example, it can reveal details of microstructures’ architecture that are difficult or impossible to visualize by SEM. TPEF microscopy is particularly attractive for this function since it is noninvasive and it does not require post-processing sample preparations. Furthermore, TPEF microscopy could be used for characterizing the homogeneity of the polymerization process that occurs during TPP. This goal could be achieved by using fluorophores that are covalently linked to the starting materials, i.e., monomers. Information regarding the dye spatial distribution within the microstructure can be obtained by TPEF microscopy, leading to a map of the polymer density.

Recently, another laser-scanning imaging technique based on a nonlinear optical phenomenon was applied for the characterization of TPP microstructures. In this study, the authors used third-harmonic generation (THG) microscopy to create high-contrast images of TPP microstructures [55]. The signal in THG microscopy stems from changes in the sample third-order nonlinear-optical susceptibility. Thus, THG microscopy permits three-dimensional imaging without the need of a fluorophore. In the characterization of TPP microstructures, THG microscopy produced the best results when the excitation light was circularly polarized.

## 10. Chemical Mapping by CARS Microscopy

Although research in TPP has reached impressive advancements such as enhanced writing resolution and wider materials availability, there are still some fundamental questions that need to be answered. For example, how does the laser or sample scanning pattern used to fabricate a specific microstructure influence its mechanical properties? How can we optimize experimental conditions to minimize microfabrication time while maintaining structural integrity? How does solvent permeation affect microstructures rigidity? At which dimensions do we have to consider the properties of the polymeric microstructures different from those of the bulk polymer?

As seen in a previous section of this review, Raman spectroscopy is an effective tool for determining the degree of polymerization in TPP microstructures. It is thus possible to correlate microstructures’ mechanical properties with the photochemistry involved during polymerization using this analytical technique. Unfortunately, Raman spectroscopy has a drawback that makes it not ideal for performing in situ and real-time characterization of TPP microstructures. Raman signals are weak and, as a consequence, they require either long acquisition times or relatively high excitation laser powers. In practice, traces with acceptable signal-to-noise ratio are obtained by averaging many spectra.

Measuring the polymerization process during TPP puts some specific demands on a potential probing technique. First, the method needs to be sensitive to the degree of chemical conversion. Second, the probing time should be compatible with the relevant timescale of TPP, which is in the range of microsecond to millisecond per voxel. Third, the probing volume needs to co-localize with the voxel addressed in TPP. These requirements allude to an optical probing technique with molecular selectivity and a sufficiently high spatial resolution.

Coherent anti-Stokes Raman scattering (CARS) microscopy can meet all these demands, and it has been shown to be a powerful diagnostic tool for characterizing chemical changes in microstructures fabricated by TPP [56,57]. Unlike spontaneous Raman scattering, CARS signals can be collected at very high acquisition rates, down to microsecond time scale. In addition, the three-dimensional probing volume in CARS microscopy resembles the voxel size in TPP. Most importantly, CARS signals can be detected on the same platform used for TPP manufacturing, offering opportunities for simultaneous writing and probing during microfabrication.

In order to generate CARS, two laser beams are focused in the sample, a pump beam at frequency ω_p_ and a Stokes beam at frequency ω_s_. Vibrational sensitivity is obtained when the difference frequency ω_p_ − ω_s_ matches the frequency of a Raman active vibrational mode of the sample. The anti-Stokes signal at ω_as_ = 2ω_p_ − ω_s_ is coherently driven by the two laser beams, resulting in coherent radiation. CARS microscopy is a four-wave mixing process and hence depends on the third-order susceptibility (χ^(3)^) of the material being investigated. Besides the vibrational contribution, χ^(3)^ also has electronic components, which results in a nonresonant response of the material. This nonresonant term is sometimes seen as a drawback for CARS microscopy since it adds a background to the imaging that is independent of the tuning of the excitation beams. Several experimental approaches have been adopted to eliminate the contribution of the nonresonant background from the CARS signal.

**Figure 11:**
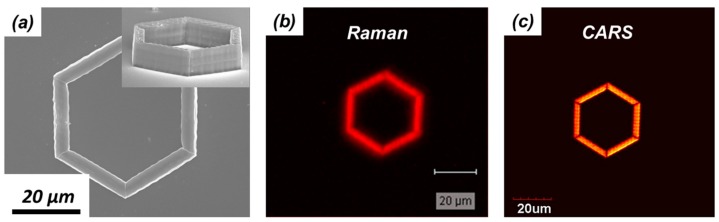
Image acquisition speed comparison between Raman confocal microscopy (**b**) and Coherent anti-Stokes Raman scattering (CARS) microscopy (**c**). Top and side SEM views of the TPP microstructure used in the test are shown in (**a**).

To demonstrate the difference between Raman and CARS signals, a simple test was performed. First, a microstructure was fabricated by TPP using an acrylic based resin (Figure 11a). Then, the microstructure was imaged using confocal Raman and CARS microscopy. The signal generated at 3000 cm^−1^ was used to build the images of the polymeric microstructure in both cases. The results of the comparison are shown in Figure 11b,c. The confocal Raman microscopy image was acquired using a microscope objective with a numerical aperture of 0.75, an excitation wavelength centered at 514.5 nm, and 35 mW of laser power. Only signal between 2800 and 3000 cm^−1^ was used. The CARS microscopy image was acquired using a microscope objective with a numerical aperture of 1.1, pump and Stokes wavelengths centered at 800 and 1050 nm, respectively. The laser average powers for the two beams were 4 mW (pump) and 10 mW (Stokes).

The confocal Raman microscopy image consists of 118 × 118 pixels and it was recorded with a pixel dwell time of around 1 s. Thus, the time required to collect all the data necessary to create Figure 11b was in excess of 4 h. The CARS microscopy image consists of 512 × 512 pixels and it was recorded with a pixel dwell time of 2 ms, resulting in an image acquisition time of just 1 s. The CARS image is sharper than the Raman image, but this can be explained by the lower number of pixels and lower spatial resolution used in the latter one. Putting aside the obvious and inevitable differences in the excitation and detection conditions used in the two imaging methods, this qualitative comparison shows that CARS microscopy is capable of acquiring an image in a time that is several orders of magnitude faster than confocal Raman microscopy. This enormous difference can be explained only by taking into consideration the fact that the CARS signal is a coherent one. Thus acquisition times and excitation powers can be lowered in CARS microscopy to values that allow for safe in situ and real-time investigation of TPP.

**Figure 12:**
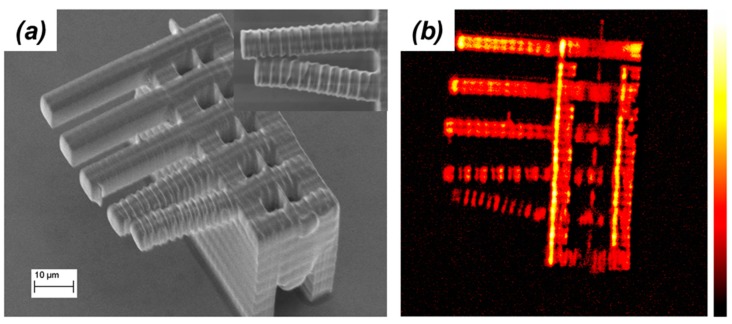
TPP microstructure imaged by (**a**) SEM and (**b**) CARS microscopy. The inset in (**a**) shows a top and magnified view of the bottom two cantilevers. The false color CARS image is made of pure resonant signal at around 3000 cm^−1^. Reproduced from [56].

Raman spectra of organic materials show strong signals around 3000 cm^−1^ due the stretching of both aliphatic and aromatic C–H bonds. Since most TPP resins contain an abundance of these modes, the first example of CARS microscopy on TPP microstructures concentrated in this spectral region. The test sample consisted of a series of hanging cantilevers 5 μm wide and 50 μm long, suspended at a height of 40 μm through a rectangular shaped tower. Figure 12a shows an SEM image of this microstructure. The five cantilevers were made following an identical procedure. 5 μm long lines (made with a single laser pass) were overlapped side-by-side for the entire length of the cantilever. The laser average power and stages velocities were kept the same during the writing. The only difference in the fabrication conditions used to create the cantilevers was the spacing between the polymeric lines that composes them. In particular, from top to bottom they were 0.1, 0.2, 0.4, 0.8, and 1.0 μm. While the first two cantilevers are straight and smooth, the last two show ridges on their surfaces. Furthermore, the shape of the last cantilever was distressed during the washing process of the unsolidified resin, highlighting its weakened structural integrity (Figure 12a inset). The surface roughness of the cantilevers can then be explained by taking into consideration the relationship between the sizes of the voxels and the spacing between the written lines.

Figure 12b shows the CARS image of the same microstructure. It is a section recorded at a height of 40 μm from the surface of the glass substrate to which the microstructure is attached. This is a chemically selective image where the strength of the signal depends exclusively on the density of C–H oscillators. It was obtained by subtracting the image recorded at 2752 cm^−1^ (which contains nonresonant contributions only) to the image recorded at 2902 cm^−1^ (which contains both resonant and nonresonant contributions). The CARS image in Figure 12b clearly shows stronger signal from the cantilevers that were fabricated by larger overlapping of polymerized lines. Furthermore, for the last two cantilevers CARS signal shows discrete jumps aligned with the laser passes used to fabricate them.

CARS signal is proportional to |χ^(3)^|^2^ and therefore strongly depends on the number of vibrational oscillators. The discontinuities in CARS signal present in Figure 9b are then a direct consequence of the density of the material. Denser is the material the higher is the concentration of C–H bonds that give rise to stronger CARS signal. Although from the SEM images we can deduce that the microstructure is a solid object, the CARS image indicates without any doubt that the density of the microstructure is not homogenous throughout the structure. Since density is related to the polymer cross-linking and polymer total conversion, CARS microscopy in the 3000 cm^−1^ region allows examining the experimental conditions used for TPP from a fabrication effectiveness point of view. For a stiffer polymer, for example, it is desirable to obtain the highest amount of cross-linking.

**Figure 13:**
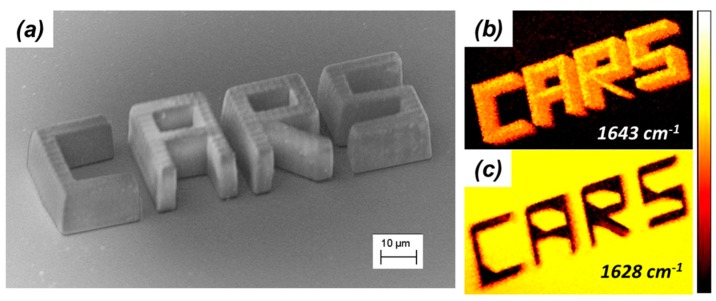
Comparison of CARS images of a TPP microstructure still immersed in the unpolymerized resin recorded at (**b**) 1643 cm^−1^ and (**c**) 1628 cm^−1^. (**a**) SEM image of the tested microstructure performed after CARS imaging (scale bar 10 µm). The relative strengths between the resin polymerized microstructure get inverted when switching probing wavenumber. Reproduced from [57].

Although performing CARS microscopy in the 3000 cm^−1^ region provides useful information on the structural integrity of three-dimensional microstructures fabricated by TPP, it would be desirable to obtain CARS images with contrast based on the chemical differences between the polymerized and unpolymerized parts of the sample (in situ characterization). This is possible by performing CARS microscopy in the fingerprint region of the TPP resins’ Raman spectra, and taking advantage of the fact that CARS signals possess highly dispersive spectral line shapes. A demonstration of this unique feature of CARS microscopy is represented in Figure 10. The word CARS was written by TPP onto a glass substrate. Each letter was 20 μm wide, 25 μm long, and 10 μm tall. An SEM image of this microstructure is shown in Figure 13a. While still immersed in the bath of unpolymerized resin, the same microstructure was imaged by CARS microscopy. Images with completely reversed contrast were obtained when the Raman shift was tuned from 1643 to 1628 cm^−1^. At 1643 cm^−1^, shown in Figure 13b, the signal from the microstructure is stronger than the signal from the unpolymerized photoresist, while at 1628 cm^−1^, shown in Figure 13c, the signal from the unpolymerized photoresist is stronger than the signal from the microstructure. Imaging with chemical contrast as shown in Figure 13, can then be employed to visually distinguish among polymerized and unpolymerized parts of microstructures fabricated by TPP.

In all the CARS examples discussed so far, the sources of the pump and Stokes beams originated from a picosecond laser and a synchronously pumped optical parametric oscillator (OPO). To acquire images in different spectral regions of the Raman spectrum of the polymeric microstructures, the output wavelengths of the OPO had to be adjusted accordingly. Although CARS microscopy by means of narrowband excitation fields permits imaging with high spectral resolution, it has the drawback of requiring different sources of light than those needed for TPP. It would be desirable to perform both TPP and CARS microscopy utilizing the same experimental setup.

To this end, the authors of one report used broadband CARS microscopy for the characterization of TPP [58]. In broadband CARS microscopy the spectral shape of the Stokes and pump beams are broad and narrow, respectively. Application of the pump and Stokes beams simultaneously excites multiple Raman transitions within the bandwidth of the Stokes pulse. Thus, broadband CARS microscopy allows gathering of a large section of the vibrational spectrum of the sample at once. Furthermore, it has been demonstrated that imaging by broadband microscopy can be efficiently performed using a single Ti:sapphire oscillator, which is a common and effective excitation source for TPP as well.

The integration of TPP and broadband CARS microscopy was achieved in the following way. The 800 nm output of the femtosecond oscillator was split in two beams. One was used for TPP, while the other was used in a device capable of generating the pump and Stokes beams. The Stokes beam was a portion of the supercontinuum generated in a photonic crystal fiber; the pump beam was the original 800 nm beam narrowed by a band-pass filter. The three laser beams were then rendered collinear by means of a series of routing optics before entering a laser scanning microscope. The pump and Stokes beams were controlled by a set of galvanometric mirrors for raster scanning to generate CARS images, while the original 800 nm laser beam was fixed and used for TPP. Fabrication was performed moving the sample in three dimensions by the aid of computer-controlled stages. The laser average power of the pump and Stokes beams were sufficiently low not to cause polymerization but high enough to generate strong CARS signals.

Thus, combining TPP and broadband CARS microscopy into one experimental setup that shares the same source of light provides the advantage of rapidly investigating TPP fabricated microstructures in situ, without the need to move the sample to another instrument. In addition, this approach makes possible monitoring TPP in real-time. Indeed, in the report mentioned above the authors provided a movie made of CARS images recorded while a 2D grid pattern was written by TPP.

## 11. Outlook and Conclusions

The dream of having an entire functional laboratory shrink down to an object that can fit in the palm of a hand is becoming more and more a reality, as critical advances in micro-manufacturing are occurring at an ever increasing speed. The driving force behind this research is the large number of applications where lab-on-a-chip devices can have a fundamental role. Examples of these applications have in part already appeared in fields such as diagnostics, genomics, and high-throughput screening [1].

In the race towards system miniaturization, the integration of TPP microstructures with microfluidics is receiving considerable attention. The three-dimensional nature of the TPP process combined with the length scale of the structures it makes permits the production of functional devices that would be difficult or impossible to make by other methods [59,60].

By means of TPP, several researchers have added uniquely designed structures to microfluidics [61]. Most of these elements are passive in their nature and they have been successfully used are microsieves, micro-overpasses, microvalves, and micromixers. Potential future applications of passive TPP elements could take advantage of its power to construct 3D structure for tissue studies on a chip [62]. There is great interest in studying drug interactions with various cell types on chips that can simulate an in vivo environment. For example, researchers have successfully grown lung cells at the interface of a dual channel microfluidic device in which one channel contains blood and the other air [63]. By periodically compressing and expanding the channel, the cells “felt” like they were in an actual lung and modeled well infections such as pneumonia. Similar platforms have been developed for many other organs including the intestines, liver, and kidneys [64]. Such platforms could stand to benefit from 3D rather than two-dimensional (2D) passive channel geometric and TPP could be important for next generation organs-on-a-chip.

It is our opinion that further research in this field should target the production of active elements by TPP based on a larger variety of physical or chemical phenomena. Some TPP microstructures used as active elements in a microfluidics system exist already. For example, magnetically and optically driven microrotors were used as chaotic mixers and pumps [65,66]. Others have used TPP of biomolecules to pattern cell growth and also to make structures that trap bacteria and use their flagella as pumps [67,68]. The combination of TPP devices coupled with living systems in microfluidics is a field with many possibilities.

Microfluidics could also enable deeper studies into the TPP reaction mechanism. For example, Raman spectroscopy could be performed on an opto-fluidic system where flow focusing is used to generate resin droplets surrounded by an inert fluid. With the aid of laser written waveguides, Raman spectroscopy can then be performed on these isolated droplets before, during, and after photopolymerization. By varying the droplet sizes and the compositions of the resin and the inert environment, such a system could provide a convenient way to isolate variables such as the diffusion of monomers or the role of oxygen. One could also imagine a Y-junction to bring into contact two solutions, one consisting of just a monomer and one with the PI only. This microfluidic system will essentially confine the polymerization volume to the interface between these two solutions where mixing happens only by diffusion.

TPP microstructures made within the channels of microfluidics could also be mechanically tested using the fluid flow. For example, a cantilever could be deflected by flowing water and the Young’s modulus could be extracted by the degree of deflection and the flow rate. Similarly, TPP cantilevers could be exposed to different solvents in a microfluidic and the deflection under a certain flow could be used to infer swelling of the polymer.

In summary, we have provided an overview of techniques used to study the photophysics of TPP and to characterize several properties of TPP microstructures. We hope this manuscript will provide the necessary information and background to the scientists and engineers interested in TPP, and inspiration to advance our knowledge of the mechanism involved in the three-dimensional microfabrication by TPP.

The advances made so far in the field of TPP metrology are the consequence of the interests of individual research groups. If the use of TPP is to move outside of academia and become a manufacturing tool, there will be a need for standards that can be used to evaluate the performance of the tools used to perform TPP. Further research in TPP metrology will have a fundamental role in developing these standards.
